# Discovery of Small Molecule KCC2 Potentiators Which Attenuate *In Vitro* Seizure-Like Activity in Cultured Neurons

**DOI:** 10.3389/fcell.2022.912812

**Published:** 2022-06-24

**Authors:** Francis J. Prael III, Kwangho Kim, Yu Du, Brittany D. Spitznagel, Gary A. Sulikowski, Eric Delpire, C. David Weaver

**Affiliations:** ^1^ Department of Pharmacology, Vanderbilt University, Nashville, TN, United States; ^2^ Vanderbilt Institute of Chemical Biology, Vanderbilt University, Nashville, TN, United States; ^3^ Department of Chemistry, Vanderbilt University, Nashville, TN, United States; ^4^ Department of Anesthesiology, Vanderbilt University School of Medicine, Nashville, TN, United States

**Keywords:** KCC, small molecule, potentiator, HTS, epilepsy

## Abstract

KCC2 is a K^+^-Cl^−^ cotransporter that is expressed in neurons throughout the central nervous system. Deficits in KCC2 activity have been implicated in a variety of neurological disorders, including epilepsy, chronic pain, autism spectrum disorders, and Rett syndrome. Therefore, it has been hypothesized that pharmacological potentiation of KCC2 activity could provide a treatment for these disorders. To evaluate the therapeutic potential of pharmacological KCC2 potentiation, drug-like, selective KCC2 potentiators are required. Unfortunately, the lack of such tools has greatly hampered the investigation of the KCC2 potentiation hypothesis. Herein, we describe the discovery and characterization of a new class of small-molecule KCC2 potentiator. This newly discovered class exhibits KCC2-dependent activity and a unique mechanistic profile relative to previously reported small molecules. Furthermore, we demonstrate that KCC2 potentiation by this new class of KCC2 potentiator attenuates seizure-like activity in neuronal-glial co-cultures. Together, our results provide evidence that pharmacological KCC2 potentiation, by itself, is sufficient to attenuate neuronal excitability in an *in vitro* model that is sensitive to anti-epileptic drugs. Our findings and chemical tools are important for evaluating the promise of KCC2 as a therapeutic target and could lay a foundation for the development of KCC2-directed therapeutics for multiple neurological disorders.

## Introduction

KCC2 (*SLC12A5*) is a K^+^-Cl^−^ cotransporter that utilizes the electrochemical K^+^ gradient to drive the efflux of Cl^−^ from cells (Payne, 1997). This activity is critical for establishing and maintaining low intracellular Cl^−^ [(Cl^−^)_i_] in neurons of the mature nervous system (Williams et al., 1999; Schulte et al., 2018). It is the resulting Cl^−^ gradient that underlies the ability of GABA_A_ and glycine receptors to mediate inhibitory neurotransmission and a healthy excitatory/inhibitory balance.

Pathologically elevated levels of [Cl^−^]_i_ in neurons are implicated in a variety of neurological disorders, including: epilepsy (Duy et al., 2019; Fukuda and Watanabe, 2019) neuropathic pain (Kahle et al., 2014a), autism spectrum disorders (Tyzio et al., 2014), and Rett syndrome (Tang et al., 2019). These pathologically high levels of neuronal [Cl^−^]_i_ are thought to cause excessive neural excitability that is a hallmark of these disorders (Schulte et al., 2018; Akita and Fukuda, 2020). During pathological states, elevated [Cl^−^]_i_ is thought to cause excess neuronal excitability through decreasing the efficacy of Cl^−^ channel effectors of fast inhibitory neurotransmission, namely GABA_A_ and glycine receptors, which use the Cl^−^ gradient generated by low neuronal [Cl^−^]_i_ to exert their inhibitory effects. Diminished fast inhibitory neurotransmission leads to pathological increases in excitatory neuronal activity (Raimondo et al., 2017). Therefore, restoring a normal [Cl^−^]_i_ through potentiation of KCC2-mediated Cl^−^ transport could counteract pathologically elevated levels of neuronal [Cl^−^]_i_ in the epilepsies and other diseases, and thus produce a therapeutic effect.

There is consistency between 1) clinical and 2) preclinical evidence supporting KCC2 potentiation as a viable therapeutic strategy in epilepsy. 1) Clinically, over a dozen loss-of-function KCC2 mutations in humans have been linked to epilepsies (Kahle et al., 2014b; Puskarjov et al., 2014; Stödberg et al., 2015; Saitsu et al., 2016; Saito et al., 2017), and resected tissue from epileptic patients has shown electrophysiological and protein expression-level abnormalities consistent with diminished KCC2 function (Huberfeld et al., 2007; Munakata et al., 2007). 2) preclinically, pharmacological (Sivakumaran et al., 2015; Kelley et al., 2016; Dzhala and Staley, 2020) or genetic (Woo et al., 2002; Silayeva et al., 2015; Chen et al., 2017; Kelley et al., 2018) inhibition of KCC2 activity exacerbates seizure-like activity across multiple epilepsy models. In contrast, genetically increasing KCC2 activity attenuates seizure-like activity in the same model systems, without overt side effects (Chen et al., 2017; Moore et al., 2018; Magloire et al., 2019). Furthermore, TrkB modulators that indirectly increase KCC2 activity restore the efficacy of phenobarbital (PB), a GABA_A_ positive allosteric modulator and antiepileptic drug, in PB-resistant seizure models (Carter et al., 2018; Kang et al., 2020; Kipnis et al., 2020). While some preliminary evidence exists linking pharmacological KCC2 potentiation to antiepileptic efficacy (Carter et al., 2018; Dzhala and Staley, 2020; Kang et al., 2020; Kipnis et al., 2020), further validation of the efficacy of KCC2 potentiation is warranted, owing to limitations with current small-molecule KCC2 potentiators.

To further evaluate if pharmacological KCC2 potentiation is antiepileptic, small molecule KCC2 potentiators with adequate potency, efficacy, and selectivity are required. Recently described KCC2 potentiators have increased the field’s understanding of KCC2 in neurological disease (Tang et al., 2019). However, these compounds have critical limitations because they 1) either act by modulating signaling pathways that have pleiotropic effects on cells (Lee et al., 2007; Yamada et al., 2016; Tang et al., 2019; Zhang et al., 2020), thereby complicating the interpretation of their effects, or 2) have some controversy surrounding their ability to potentiate KCC2 (Gagnon et al., 2013; Cardarelli et al., 2017; Gagnon et al., 2017). Therefore, discovery of new, selective KCC2 potentiators would greatly benefit the field.

Herein we describe the discovery of a new class of small-molecule KCC2 potentiator with a unique mechanism-of-action. We demonstrate that the compounds in this class attenuate seizure-like neuronal hyperactivity in cortical neuronal-glial co-cultures providing supporting the hypothesis that pharmacological KCC2 potentiation alone is sufficient to attenuate neuronal hyper-excitability.

## Materials and Methods

### HEK-293 Cell Culture

HEK-293 cells were cultured up to 80%–90% confluence in T75 flasks (TPP) containing HEK-293 medium [α-MEM (Corning) supplemented with 10% (v/v) fetal bovine serum (FBS) (Gibco), 1x glutagro (Corning)] and appropriate antibiotics for selection (vide infra) at 37°C and 5% CO_2_. Cells were passaged every 3–4 days using TrypLE (Gibco) up to a maximum of 20 passages.

### Rat Husbandry

Timed-pregnant Sprague Dawley dams were purchased from Taconic Biosciences. Experiments involving animals were approved by and adhered to the guidelines of the Vanderbilt Institutional Animal Care and Use Committee.

### Antibodies for Western Blotting and Immunofluorescence

Primary antibodies used for Western blotting were: 1:1,000 rabbit Anti-K^+^-Cl^−^ cotransporter (KCC2) Antibody (MilliporeSigma 07-432) and 1:500 mouse Transferrin Receptor Monoclonal Antibody (H68.4) (ThermoFisher Scientific 13-6800). Secondary antibodies used for Western blotting were: 1:15,000 goat anti-Rabbit IRDye 800CW (LI-COR 926-32211) and 1:10,000 goat anti-Mouse IgG IRDye 680RD (LI-COR 926-68070). Primary antibodies used for immunofluorescence were: 1:100 mouse anti-KCC2/SLC12A5 Antibody (S1-12) (Novus Biologicals NBP2-59337) and 1:50 rabbit anti-GAD65/GAD67 (Thermo Fisher Scientific PA5-36080). Secondary antibodies used for immunofluorescence were: 1:500 goat anti-Mouse IgG IRDye 680RD (LI-COR 926-68070) and 1:500 Goat anti-Rabbit Alexa Fluor 555 Secondary Antibody (ThermoFisher A-21428). Cell nuclei were labeled using Hoechst 33,342 at 1 μg/ml.

### Molecular Cloning

The most widely expressed KCC2 isoform in the adult mammalian brain (KCC2b) was subcloned from the pCITF-KCC2 vector (Addgene plasmid #61404) into pcDNA4/TO (ThermoFisher Scientific) between the AflII and NotI restriction sites using Gibson Assembly (New England Biolabs) for inducible expression of KCC2b. SuperClomeleon (Grimley et al., 2013) was subcloned into the pENTR1A no ccDB (w48-1) (Addgene #17398) Entry Vector (Campeau et al., 2009) using Gibson Assembly. The SuperClomeleon ORF was subsequently recombined into the pLenti CMV Puro DEST (w118-1) (Addgene #17452) destination vector for lentiviral transduction and constitutive SuperClomeleon expression using LR Clonase II Enzyme Mix (ThermoFisher Scientific). The SuperClomeleon-pLenti-CMV-puro vector was propagated in NEB® Stable Competent *E. coli* (New England Biolabs) to prevent homologous recombination of long terminal repeats. All sequences were verified by Sanger Sequencing (GenHunter) before cell line generation.

### Polyclonal Cell Line Generation for the Cl^−^ Flux Assay

Two polyclonal cell lines were created for use with the Cl^−^ flux assay 1) one expressing KCC2 and 2) one lacking KCC2 expression. 1) Generation of a KCC2-expressing polyclonal cell line: to allow for inducible expression of KCC2, T-REx-293 cells (ThermoFisher Scientific) seeded at 40% confluence in a T75 flask were transfected with the pcDNA4/TO-KCC2 construct using FuGENE6 (Promega), according to the manufacturer’s instructions. One day after transfection, the cells were treated with 5 μg/ml blasticidin, to select for the tetracycline repressor protein in T-REx-293 cells, and 250 μg/ml zeocin, to select for KCC2. Cells remained under selection for 2 weeks to ensure stable KCC2 expression. To generate a stable T-REx-293-KCC2-SuperClomeleon cell line, SuperClomeleon-containing lentivirus was produced in HEK-293T cells through transfection of the pLenti-CMV-puro-SuperClomeleon transfer vector, the pCMV-VSV-G (Addgene #8454) envelope plasmid, and the pMDLg/pRRE (Addgene #12251) and pRSV-Rev (Addgene #12253) packaging plasmids using FuGENE6. The T-REx-293-KCC2 polyclonal cell line was then seeded at 40% confluence in a T75 flask and transduced with SuperClomeleon-containing lentivirus as described in (Campeau et al., 2009). One day after transduction, the virus-containing medium was removed, and the cells were washed with fresh HEK-293 medium. Cells were treated with 5 μg/ml blasticidin, 250 μg/ml zeocin, and 3 μg/ml puromycin roughly 24 h after removal of the virus to generate the polyclonal T-REx-293-KCC2-SuperClomeleon cell line. 2) Generation of a control cell line lacking KCC2 expression: The T-REx-293-SuperClomeleon polyclonal cell line was generated by transducing T-REx-293 with SuperClomeleon-bearing lentivirus as described above. A clone was selected whose YFP and CFP fluorescence levels matched those of uninduced 4p2.F7 cells used for screening (see below) to control for baseline SuperClomelon expression.

### Monoclonal Cell Line Generation for HTS

The T-REx-293-KCC2-SuperClomeleon polyclonal cell line was cell-sorted based on highest YFP and CFP fluorescence into individual wells of a 96-well, TC-treated plate (Corning) using a BD FACSAria IIIu. After allowing the cells to proliferate to near confluence in the presence of antibiotic selection, the ∼200 resulting monoclonal cell lines were screened using the Cl^−^ flux assay (described below) ± 100 µM KCC2 potentiator N-Ethylmaleimide (NEM) (MilliporeSigma) and ± KCC2 induction with 1 µM tetracycline (MilliporeSigma). The six cell lines having the highest KCC2 induction-dependent NEM activity, as determined by the highest Area Under the Curve (AUC) of the YFP/CFP ratio in treated vs. untreated cells, were then tested for 1) suitability for HTS by Z′ value calculation (see below), and 2) for predicted KCC2 pharmacology by testing a panel of known KCC2 modulators (Supplementary Figure S1A). The cell line, 4p2.F7, which had the highest Z’ value and exhibited pharmacology consistent with KCC2 expression was subsequently used for HTS.

### Cl^−^ Flux Assay

**FIGURE 1:**
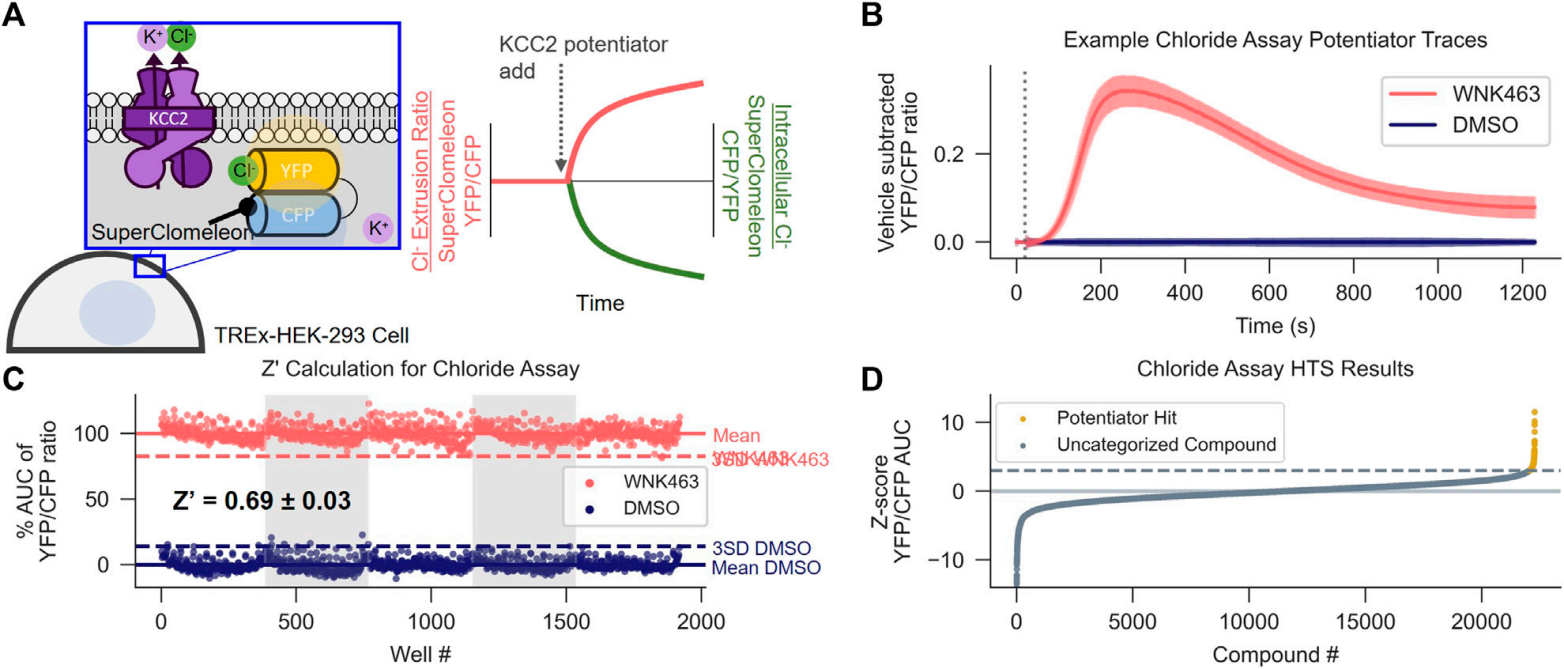
Discovery of KCC2 Potentiators by High-throughput Screening. **(A)** Cl^−^ flux assay schematic and data normalization. KCC2 activity is approximated by measuring intracellular Cl^−^ using HEK-293 cells overexpressing KCC2 and the Cl^−^ sensor SuperClomeleon. Data are normalized as a YFP/CFP ratio, such that an increase in KCC2 activity registers as an increase in value. **(B,C)** Calculation of Z′ for the Cl^−^ flux assay. **(B)** Vehicle control subtracted and averaged traces from the Cl^−^ flux assay used for Z′ calculation. Cells were treated with either the indirect KCC2 potentiator WNK463 or DMSO control. Error bars represent standard deviation of the mean (SD). **(C)** Percent Area Under the Curve (AUC) of Cl^−^ flux assay traces in **(B)**. The mean AUC (solid line) and three x SD away from mean AUC (dotted line) are displayed for each treatment (*n* = 192). **(D)** Results of screening approximately 23,000 unique compounds at a concentration of 10 µM in the Cl^−^ flux assay. Data are presented as Z-scores relative to vehicle control values on each plate. A Z-score of 3 (dotted line) was used as a cut off for separating potentiator hits (gold) from uncategorized compounds (gray). All experiments were performed at 37°C.

The Cl^−^ flux assay functioned by monitoring [Cl^−^]_i_ levels via a the genetically encoded Cl^−^ sensor, SuperClomeleon (Grimley et al., 2013), and, after compound treatment, using the reported changes in [Cl^−^]_i_ as a surrogate for changes in KCC2 Cl^−^ transport activity. The SuperClomeleon Cl^−^ sensor functions by FRET between a Cl^−^-sensitive YFP, which is quenched upon Cl^−^ binding, and a Cl^−^-insensitive CFP, which is used as a control for SuperClomeleon expression. The corresponding changes in FRET ratio are then used to quantify changes in [Cl^−^]_i_. The day before the experiment, HEK-293 cells were counted and plated at 20,000 cells/well in black-walled, amine-coated, 384-well plates (Corning) at 20 µl/well in HEK-293 medium supplemented with 10 ng/ml tetracycline to induce KCC2 expression. After incubation for approximately 24 h at 37°C and 5% CO_2_, cell culture medium was removed by centrifugation in the Blue Washer (BlueCatBio) under the GentleSpin setting, and 20 µl/well Assay Buffer [1x Hank’s Buffered Salt Solution (ThermoFisher) + 20 mM HEPES (Corning) (pH 7.3)] was added to each well. Compound plates were made in polypropylene, v-bottom, 384-well plates (Greiner) with compounds dissolved at 2x relative to the desired concentration in Assay Buffer; dimethyl sulfoxide (DMSO) concentrations were kept below 0.8% (v/v) final. Cell plates were incubated at 37°C within the Panoptic plate reader (WaveFront Biosciences) for at least 30 min prior to the beginning of the run to allow for temperature equilibration to 37°C. To measure Cl^−^ flux, fluorescence was recorded at 1 Hz [excitation at 440/40 nm and emission alternating between 480/17 nm (CFP) and 536/40 nm [YFP FRET]) for 20 s, then 20 µl/well of the 2x compound solution was added and fluorescence was recorded for an additional 20 min. For data analysis, a YFP/CFP FRET ratio was calculated by taking the YFP fluorescence intensity value and, to account for the temporal offset between CFP and YFP recordings, interpolating CFP values to match the YFP timepoints. The YFP values were then divided by the interpolated CFP values to generate a YFP/CFP FRET ratio to quantify Cl^−^ extrusion (Figure 1A). The FRET ratio traces were subsequently normalized by dividing each fluorescence trace by the trace’s average FRET ratio before compound addition. To quantify Cl^−^ flux over the course of the run, average vehicle control-treated traces were subtracted from each trace to reveal the signal dependent on presence of an active test compound, and AUC for each FRET trace was calculated using the trapezoidal rule. For cotreatment experiments, compounds were sourced from the following distributors: Go6983 (Tocris), WNK463 (Selleckchem), Bumetanide (MilliporeSigma), and VU0463271 (Tocris). All other compounds were either synthesized in house (see Synthesis sections below) or purchased from Life Chemicals Inc.

### Z’ Calculation

Z’ values were calculated as in (Zhang et al., 1999) using the following formula:

$${\text{Z}}^{\prime }=1-\left(\left(3\sigma \_\left(\text{c}+\right)\right)+\left(3\sigma \_\left(\text{c}-\right)\right)/\left(\left|\mu \_\left(\text{c}+\right)-\mu \_\left(\text{c}-\right)\right|\right)\right)$$

Where *σ*_(c+) is the standard deviation (SD) of positive control (KCC2 potentiator)-treated wells, 3*σ*_(c−) is the SD of the vehicle-treated wells, μ_(c+) is the mean of positive control-treated wells, and *μ*_(c−) is the mean of the positive control-treated wells.

### Thallium (Tl^+^) Influx Assay

The day before the experiment, HEK-293 derived cell lines were resuspended in HEK-293 medium supplemented with 100 ng/ml tetracycline to induce KCC2 expression, counted, and were plated at 20,000 cells/well in black-walled, amine-coated, 384-well plates at 20 µl/well. On the day of the experiment, a 5x Tl^+^ stimulus solution [125 mM sodium bicarbonate, 12 mM thallium sulfate, 1 mM magnesium sulfate, 1.8 mM calcium sulfate, 5 mM glucose, and 10 mM HEPES (pH 7.3)] was prepared and pipetted into a polypropylene, v-bottom, 384-well plate. Compounds were prepared at a 2x concentration in Assay Buffer in a separate polypropylene, v-bottom, 384-well plate, with a final DMSO concentration below 0.8% (v/v). The stimulus and 2x compound plates were sealed and incubated for at least 30 min at 37°C within the Panoptic plate reader before assaying. After approximately 24 h of induction, the cells were loaded with the Tl^+^-sensitive dye, Thallos (ION Biosciences): the cell culture medium in the cell plate was removed and replaced with 20 µl/well Assay Buffer containing 2.5 μg/ml Thallos-AM, and cells were dye-loaded for 45 min at 37°C. After dye loading, the dye-loading solution was removed and replaced with 20 µl/well Assay Buffer pre-warmed to 37°C. The cell plate was then incubated for 10 additional minutes at 37°C in the Panoptic before assaying. After incubation, fluorescence intensity values were recorded at 1 Hz (482/35 nm excitation and 536/40 nm emission) for 10 s prior to compound addition. 20 µl/well of 2x compound solution was added to the cells and incubated for 10 min followed by the addition of 10 µl/well of the 5x Tl^+^ stimulus solution and an additional 1 min of data collection. Data for each well were normalized by dividing data at each time point for a given well by the average of its own pre-compound addition baseline fluorescence (F/F_0_) and Tl^+^ influx was quantified by the maximum fluorescence after Tl^+^ stimulus addition. The T-REx-293-KCC2 monoclonal cell line, TK2D2, and untransfected T-REx-293 cells were used for all Tl^+^ influx experiments. The TK2D2 cell line was generated from the T-REx-293-KCC2 polyclonal cell line described in the “Polyclonal Cell Line Generation for the Cl^−^ Flux Assay” section by serial dilution and subsequent clonal selection based on maximum Tl^+^ influx that was sensitive to the KCC2 inhibitor VU0463271.

### High-Throughput Screening

Eighty nanoliters of 10 mM (nominal) test compound dissolved in DMSO was transferred from the Vanderbilt Institute of Chemical Biology (VICB) Discovery Collection to 384-well, v-bottomed, polypropylene plates (Greiner) using an Echo 555 Acoustic Liquid Handler (Labcyte) and diluted to a concentration of 20 µM by addition of 40 µl/well Assay Buffer using a Multidrop Combi Reagent Dispenser (ThermoFisher Scientific). The compound plates were mixed on a plate shaker (ThermoFisher Scientific) for at least 2 min, sealed and bath sonicated (Branson) for approximately 1 min. Compound plates and cell plates were incubated at 37°C for at least 30 min before assaying. The 4p2.F7 T-REx-293-KCC2-SuperClomeleon cell line was prepared and assayed at 37°C, at final compound concentration of 10 µM (nominal), as described in the Cl^−^ flux assay section. Z-scores were calculated relative to the YFP/CFP ratio AUCs of vehicle-treated wells on a plate-by-plate basis. Potentiator Hits were defined as compounds with a Z-score > 3. The first 9,000 compounds were screened at random from the VICB Discovery Collection. The final 14,000 compounds were iteratively screened, in batches of approximately 3,000 compounds, to enrich for compound classes which could accommodate chemical variation while still potentiating KCC2-dependent Cl^−^ extrusion in four steps. First, hits were selected and re-tested in the Cl^−^ flux assay, with or without KCC2 expression, to gauge reproducibility and KCC2-dependence. Second, hits were tested in the Tl^+^ influx assay to test if their activity was consistent across disparate assays of KCC2 ion transport. Third, hits that exhibited reproducible and consistent activity at KCC2 were enriched in the next round of screening by selecting plates from the compound library containing structurally similar compounds, together with random compounds to continue a broad sampling of the chemical library throughout the screen. Compounds of similar structure were selected using chemical similarity searching in Python, using the RDKit Library (RDKit.org): chemical fingerprints for the entire compound library were generated using RDKit fingerprints, and similarity was quantified between hits and the rest of the VICB Discovery Collection by Tanimoto coefficient calculation. The highest Tanimoto coefficients were interpreted as the most chemically similar compounds. Fourth, compound plates containing the enriched compound set was then screened, and the process repeated after hit determination.

### Potassium (^83^Rb^+^ Tracer) Influx Assay

Wild-type or KCC2-expressing HEK-293 cells were grown in 10-cm dishes in DMEM/F12 medium supplemented with 5% (v/v) fetal bovine serum and 1% (v/v) penicillin/streptomycin. For K^+^ influx experiments, cells were plated (2 ml/dish) for 2 h in 35-mm, poly-L-Lysine (0.1 mg/ml, Sigma)-coated dishes. Medium was then aspirated and replaced with 1 ml of the appropriate buffer (below) for a 10 min preincubation period. For KCC2-mediated ^83^Rb^+^ influx measurements, cells were rinsed and incubated with a Na^+^-free solution containing 132 mM N-methylglucamine (NMDG)-Cl, 5 mM KCl, 2 mM CaCl_2_, 0.8 mM MgSO_4_, 1 mM glucose, 5 mM HEPES, pH 7.4 with NMDG. For NKCC1-mediated K^+^ influx measurements, the solution contained 132 mM NaCl, 5 mM KCl, 1 mM CaCl_2_, 0.8 mM MgSO_4_, 1 mM glucose, 60 mM sucrose, 5 mM HEPES, pH 7.4 with NaOH. Following the preincubation period, the medium was aspirated and replaced with identical solutions containing 200 μM ouabain and 0.25 μCi/ml ^83^Rb^+^. After three rapid washes in ice-cold solution, the cells were lysed with 0.5 ml 0.5 N NaOH for 1 h then neutralized by adding 0.25 ml glacial acetic acid. Aliquots were then collected for protein assay (Biorad) and β-scintillation counting. K^+^-influx were calculated based on ^83^Rb^+^ uptake and expressed in pmole K^+^/mg protein/min.

### Surface Biotinylation

The surface biotinylation protocol was based on previously published work by Friedel et al. (2017), with modifications to accommodate compound treatment. The day before the experiment, 4p2.F7 T-REx-293-KCC2-SuperClomeleon cells were plated in 10 cm, TC-treated, cell-culture dishes containing HEK-293 medium supplemented with 1 μg/ml tetracycline to achieve 90% confluence during surface biotinylation. After approximately 24 h, cells were washed in Assay Buffer and treated with test compounds dissolved in Assay Buffer that had been warmed to 37°C. After 15 min at 37°C, the cells were washed once in Assay Buffer, and incubated with the compound-containing Assay Buffer solution supplemented with 0.4 mg/ml EZ-link Sulfo-NHS-SS Biotin (Pierce 89,881) at 20°C for 30 min. The biotinylation reaction was subsequently quenched using Phosphate-Buffered Saline (PBS)/Ca/Mg [PBS containing 0.1 mM CaCl_2_ and 1 mM MgCl_2_ (pH 7.3)] that had been supplemented with 100 mM lysine and adjusted to final osmolality of 300 mOsm with H_2_O. Cells were then washed three times in ice-cold PBS/Ca/Mg and harvested by cell scraping. Cell pellets were generated by centrifugation at 500x g for 2 min, the supernatant solution was removed by aspiration, and the cells were transferred in ice-cold Lysis Buffer [50 mM NaCl, 1% (v/v) Triton X-100, 0.5% (v/v) deoxycholate, 0.1% (v/v) SDS, 50 mm Tris-HCl, 10 mM iodoacetamide, and protease inhibitors (ThermoFisher Scientific 78430) (pH 8)] to a microcentrifuge tube, and lysed with gentle agitation at 4°C for 30 min. Lysates were centrifuged at 1,200x g at 4°C for 5 min, and the clarified lysates were transferred to a new microcentrifuge tube. Protein concentrations were determined by BCA assay (Pierce) using BSA (MilliporeSigma) as a standard. Lysate containing 20 µg of protein were set aside at 4°C overnight, for use as a control of total protein levels, and lysate containing 400 µg of protein was incubated with streptavidin-conjugated agarose (Pierce 89,881) overnight at 4°C with gentle agitation. The next day, the agarose beads with bound biotinylated proteins were washed 3 times in ice-cold lysis buffer, and one time in ice-cold PBS/Ca/Mg supplemented with 10 mM iodoacetamide. Protein was eluted from agarose beads in 40 µl of Loading Buffer [65.8 mM Tris-HCl, 26.3% (w/v) glycerol, 2.1% (w/v) SDS, 5% (v/v) β-mercaptoethanol (pH 7)] pre-heated to 95°C. The 20 µg total protein sample (above) and 40 µl of the eluted protein from the streptavidin beads were then loaded onto an SDS-PAGE gel for Western blotting and quantification as described below.

### SDS-PAGE and Western Blot

HEK-293 cells were lysed and protein concentrations quantified as described in the Surface Biotyinlation section. 20 µg of lysate, unless otherwise specified, was diluted 1:1 in Loading Buffer that was preheated to 95°C, immediately loaded onto a NuPAGE Bis-Tris 4%–12% (w/v) SDS-PAGE gel (ThermoFisher Scientific) and separated using the XCell SureLock Mini-Cell system (ThermoFisher Scientific). Proteins were transferred to a PVDF membrane using the iBlot2 transfer system (ThermoFisher Scientific), incubated in Intercept Blocking Buffer (LI-COR) for 1 h at room temperature to inhibit non-selective antibody binding, and incubated in primary antibody overnight. The blots were washed 3 times in Tris-Buffered Saline-Tween (TBS-T) [150 mM NaCl, 25 mM Tris base, 0.05% (v/v) Tween 20 (pH 7.4)] for 5 min each time, incubated with secondary antibody for 1 h at room temperature, washed 3 times in TBS-T, and rinsed in TBS. All blots were imaged using the Odyssey CLx system (LI-COR) and quantified using the Image Studio acquisition software (LI-COR). Consistent with previous reports, KCC2 had a multiband pattern (6). We interpreted the lower band at approximately 120 kDa, the predicted molecular weight for full length KCC2, as monomeric KCC2. We interpreted the bands at approximately 240 kDa and above as dimeric- and higher-order oligomers of KCC2. For quantification of the amount of KCC2 in each sample, values for monomeric and oligomeric bands were pooled. For surface biotinylation experiments, the amount of endogenous transferrin receptor (TfR) expression was used to normalize for loading differences in the total- and surface-fractions of KCC2. For surface/total protein quantification, the KCC2 signal from surface and total fractions were normalized to their respective TfR levels, and then the normalized KCC2 surface values were divided by normalized KCC2 total values.

### Neuronal-Glial Co-Culture

Brain cortices were isolated from E18 Sprague Dawley rat embryos and placed in ice-cold PBS and prepared as described in (Pacico and Mingorance-Le Meur, 2014). Cortices were dissociated in 0.25% (w/v) Trypsin (Gibco) at 37°C for 20 min. The trypsin reaction was quenched by addition of neurobasal complete medium (NbC)[1x Neurobasal (Gibco), 1x B-27 supplement (Gibco), 1x glutamax (Gibco), 100 U/ml penicillin-streptomycin (Gibco)] supplemented with 10% (v/v) horse serum (NbC + HS) (Gibco). The tissue was then pelleted at 100x g for 5 min, the supernatant solution was removed by aspiration, and a single-cell suspension was generated by trituration in NbC + HS medium. The cells were then counted, diluted to 500 cells/µl in NbC + HS, and 100 µl of this suspension was plated in black-walled, poly-L-lysine-coated, 96-well plates (Greiner) for a final density of 50,000 cells/well. The neuronal-glial cocultures were then incubated at 37°C and 5% CO_2_ for 4 h, and the serum-containing NbC + HS medium was then replaced with serum-free NbC medium. The neuronal-glial co-cultures were maintained until at least 14 days *in vitro* (DIV) at 37°C and 5% CO_2_ before assaying. Half of the medium was exchanged with fresh NbC medium every 3–4 days.

### Synchronized Neuronal Ca^2+^ Oscillation Assay

Neuronal-glial co-cultures were assayed between 14 DIV and 18 DIV using a protocol adapted from (Pacico and Mingorance-Le Meur, 2014). The medium was removed from the co-cultures and the co-cultures were loaded with Fluo-8 (AAT Bioquest) by incubation in 90 µl/well dye-loading solution [1x HBSS (Corning), 20 mM HEPES (MilliporeSigma), 1 μg/ml Fluo-8 AM, 1 mM probenecid, 3 mg/ml BSA (MilliporeSigma) (pH 7.3)] for 30 min at 37°C. The dye-loading solution was then removed and replaced with 180 µl/well Neuronal Assay Buffer [1x Ca^2+^- and Mg^2+^-free HBSS (Corning) + 2 mM CaCl_2_ + 3 mM MgCl_2_ + 20 mM HEPES (pH 7.3)] that was pre-warmed to 37°C. The co-cultures were then immediately loaded into the Panoptic plate reader and assayed. Cultures were continuously imaged at 5 Hz (482/35 nm excitation and 536/40 nm emission) at 37°C throughout the run. After a 3-min reading to establish a Ca^2+^ oscillation baseline, 20 µl of a 100x compound solution was added to the co-cultures using the Panoptic. Co-cultures were subsequently imaged for 12 min to quantify the effect of compounds on the Ca^2+^ oscillation rate. Ca^2+^ oscillation rates were quantified using Python. Our data analysis workflow consisted of: 1) correction for baseline drift, 2) peak counting, and 3) oscillation rate normalization. 1) correction for baseline drift: fluorescent traces were corrected by calculating a smoothened version of each trace and then subtracting the smoothened trace from the original trace. Traces were smoothened by a mean filter generated by iterative convolutions of a 100-frame and a 200-frame averaging kernel over the timeseries data; afterward, the smoothened trace was subtracted from the overlapping region of the original trace. 2) peak counting: peaks were counted separately in the pre-compound addition and post-compound addition segments by counting peak-to-peak local maxima using a manually set threshold value and minimum distance between peaks to prevent over/under counting of peaks. 3) oscillation rate normalization: oscillation rates were calculated by dividing Ca^2+^ oscillation counts by time. Lastly, post-compound addition oscillation rates were normalized as a percentage of the baseline oscillation rate to yield a “% baseline oscillation rate.” All edge wells in the 96-well plate (Rows A and H, together with columns 1 and 12) were omitted from data analysis owing to differences in signal observed in those wells relative to the rest of the plate.

### Immunofluorescence

Immunofluorescence staining of cortical cocultures was performed after 14 DIV as described above for Ca^2+^-imaging experiments. NbC medium was removed and cells were washed 3 times with PBS and fixed with 4% (w/v) paraformaldehyde in 1x HBSS for 20 min at room temperature. Cells were then washed with PBS and subsequently permeabilized with 0.3% (v/v) Triton X-100 (MilliporeSigma) in PBS for 15 min. Permeabilized cells were blocked in 10% (v/v) bovine serum albumin (BSA) (MilliporeSigma) in PBS for 45 min cells were washed 3 times with PBS. Cells were treated with mouse anti-KCC2/SLC12A5 antibody (S1-12) and rabbit anti-GAD65/GAD67 in 1% BSA in PBS at 4°C overnight. Cells were washed 3 times with PBS prior to being incubated with goat anti-mouse and goat anti-rabbit AF555 secondary antibodies for 45 min at room temperature. Cells were washed 3 times with PBS and stained with Hoechst (1:5,000, ThermoFisher Scientific, H3570). Images were recorded using the ImageXpress Micro XLS system (Molecular Devices).

### Statistical Analyses

All reported data were drawn from at least three independent experimental replicates. Statistical analyses were conducted in R and Python. All tests with a two-sided *p* < 0.05 were considered statistically significant. Assumptions of normality were tested using the Shapiro-Wilk test. For two samples of normally distributed data, the Welch’s unequal variances t-test was used for hypothesis tests using independent data, and the paired t-test was used for dependent data. To test if one, normally distributed sample was statistically significant from a single value, a one sample t-test was used. For two samples of data that were not necessarily normally distributed, the Mann-Whitney U test was used. For hypothesis testing of linear regression models containing normally distributed inputs, the Wald test was used with a t-distribution of the test statistic.

### Compound Synthesis General Procedure

All non-aqueous reactions were performed in flame-dried or oven-dried round-bottomed flasks under an atmosphere of argon. Stainless steel syringes or cannula were used to transfer air- and moisture-sensitive liquids. Reaction temperatures were controlled using a thermocouple thermometer and analog hotplate stirrer and monitored using liquid-in-glass thermometers. Reactions were conducted at room temperature (approximately 21°C–23°C) unless otherwise noted. Flash column chromatography was conducted using silica gel 230–400 mesh. Reactions were monitored by analytical thin-layer chromatography, using Silica Gel 60 F254 glass-backed pre-coated silica gel plates (MilliporeSigma). The plates were visualized with UV light (254 nm) and stained with potassium permanganate or p- anisaldehyde-sulfuric acid followed by charring. Yields were determined by weight of isolated, spectroscopically pure compounds.

### Materials for Compound Synthesis

Solvents and chemicals were purchased from Sigma-Aldrich, Acros Organics, TCI and/or Alfa Aesar and used without further purification. Solvents were purchased from Fisher Scientific. Dry dichloromethane (DCM) was collected from an MBraun MB-SPS solvent system. Dichloroethane (DCE) was distilled from calcium hydride and stored over 4 Å molecular sieves. Triethylamine, N,N-dimethylformamide (DMF) and DMSO were used as received in a bottle with a Sure/Seal. N,N-diisopropylethylamine was distilled from calcium hydride and stored over KOH. BF_3_·diethyl ether was distilled prior to use from calcium hydride. Deuterated solvents were purchased from Cambridge Isotope Laboratories.

### Instrumentation for Compound Synthesis

Preparative reverse-phase HPLC (Gilson) was performed using a Phenomenex Gemini column (5 micron, 110 Å, 50 mm × 21.20 mm, flow rate 30 ml/min) with UV/Vis detection. Low-resolution mass spectra were obtained on an Agilent 1200 series system with UV detection at 214 and 254 low-resolution mass spectra were obtained on an Agilent 6130 single quad mass spectrometer with electrospray ionization (ESI) in positive mode. LC-MS experiments were performed with the following parameters: Accucore C18 column 2.6 μm, 2.1 mm × 30 mm column) at 40°C; an acetonitrile (ACN)/water with 0.1% (v/v) trifluoroacetic acid gradient 7%–90% (v/v) ACN (method 1) for 1.5 min and 40%–90% (v/v) ACN (method 2) for 1.5 min; flow rate of 1.5 ml min-1. 1H NMR spectra were recorded on Bruker 400 or 600 MHz spectrometers and are reported relative to internal chloroform (1H, *δ* 7.26), methanol (1H, *δ* 3.31), and DMSO (1H, *δ* 2.50). Data for 1H NMR spectra are reported as follows: chemical shift (*δ* ppm), multiplicity (s, singlet, d, doublet, t, triplet, dd, doublet of doublet, ddd, doublet of doublet of doublet, m, multiplet, br, broad), coupling constants (Hz), and integration. 13C NMR were recorded on Bruker 100 or 150 MHz spectrometers and are reported relative to internal chloroform (13C, *δ* 77.1), methanol (MeOH) (13C, *δ* 49.2), and DMSO (13C, *δ* 40.3).

### Methyl 4-(P-Tolylthio)Butanoate 3

A mixture of methyl 4-bromobutanoate (2.19 g, 12.07 mmol), 4-methylbenzenethiol (1.0 g, 8.05 mmol), and K_2_CO_3_ (2.22 g, 16.10 mmol) in ACN (24 ml) was stirred for 3 h and solvent was removed in vacuo. The crude residue was dissolved in ethylacetate (EtOAc) (30 ml), washed with saturated NH_4_Cl solution (30 ml), extracted with EtOAc (3 ml × 30 ml), and dried over MgSO_4_. The solvent was removed under reduced pressure and the residue was purified by column chromatography (0%–30% (v/v) hexane/EtOAc gradient) to afford sulfide 3 as a yellow oil (2.46 mg, 95%). LCMS (ESI) Rt 1.14 min, m/z: 225.2 [M + H]+.

### Methyl 4-Tosylbutanoate 4

To a solution of ester 3 (1.0 g, 4.46 mmol) in MeOH/tetrahydrofuran (THF) (22 ml/66 ml) was added a solution of Oxone (31.2 ml, 1.0 M in water). The reaction mixture was stirred for 20 h and concentrated in vacuo. The crude mixture was diluted with water (50 ml), extracted with EtOAc (3 ml × 50 ml), and dried over MgSO_4_. The solvent was removed under reduced pressure and the residue was purified by column chromatography (0%–50% (v/v) hexane/EtOAc gradient) to afford sulfone 4 as a yellow oil (0.66 g, 58%). LCMS (ESI) Rt 0.86 min, m/z: 257.1 [M + H]+.

### 4-Tosylbutanoic Acid 5

To a solution of sulfone 4 (0.66 g, 2.57 mmol) in dioxane/methanol (10 ml/5 ml) was added NaOH (6.43 ml, 1.0 M solution). The reaction mixture was stirred for 3 h at 40°C, acidified to pH 2 with HCl (1.0 N solution), extracted with EtOAc (3 ml × 20 ml), and dried over MgSO4. The crude product was used without further purification. 1H NMR (DMSO-d6, 400 MHz) *δ* 7.76 (d, J = 8.0 Hz, 2H), 7.48 (d, J = 8.0 Hz, 2H), 3.32–3.27 (m, 2H), 2.43 (s, 3H), 2.33 (t, J = 7.2 Hz, 2H), 1.75–1.67 (m, 2H); LCMS (ESI) Rt: 0.72 min, m/z: 243.1 [M + H]+.

### N-(4-Methylbenzo[d]Thiazol-2-yl)-4-Tosylbutanamide 6

To a solution of 4-tosylbutanoic acid 5 (100 mg, 0.61 mmol) in DMF (3 ml) was added N,N-Diisopropylethylamine (DIPEA) (0.43 ml, 2.44 mmol), followed by 1-[Bis(dimethylamino)methylene]-1H-1,2,3-triazolo[4,5-b]pyridinium 3-oxide hexafluorophosphate (HATU, 0.35 g, 0.92 mmol). After stirring for 30 min at 0°C, 4-methylbenzo[d]thiazol-2-amine (148 mg, 0.61 mmol) was added to reaction mixture. The reaction mixture was stirred 12 h, diluted with water (5 ml), and resulting precipitate collected and washed with water (3 ml × 10 ml). Amide 6 was obtained as a white solid product (174 mg, 73%) by filtration. 1H NMR (DMSO-d6, 400 MHz) *δ* 7.78 (dd, J = 8.8, 8.4 Hz, 3H), 7.48 (d, J = 8.0 Hz, 2H), 7.24 (d, J = 8.0 Hz, 1H), 7.19 (t, J = 8.4 Hz, 1H), 3.37–3.33 (m, 2H), 2.60 (t, J = 7.2 Hz, 2H), 2.56 (s, 3H), 2.42 (s, 3H), 1.86 (q, J = 7.2 Hz, 2H); LCMS (ESI) Rt 1.09 min, m/z: 389.3 [M + H]+.

### N-(2-(Dimethylamino)Ethyl)-N-(4-Methylbenzo[d]Thiazol-2-yl)-4-Tosylbutanamide (VU0500469)

To a solution of N-(4-methylbenzo[d]thiazol-2-yl)-4-tosylbutanamide (50 mg, 0.128 mmol) in THF (3 ml) was added sodium bis(trimethylsilyl)amide (0.384 ml, 1 M solution in THF) at 0°C. After stirring for 10 min a solution of 2-bromo-N,N-dimethylethan-1-amine (60 mg, 0.26 mmol) in THF (0.8 ml) was added, the mixture was allowed come to room temperature, stirred for 12 h, and quenched with saturated NH_4_Cl solution (10 ml). The quenched reaction was extracted with EtOAc (3 ml × 10 ml), and dried over MgSO_4_. The solvent was removed under reduced pressure and the residue was purified by column chromatography (0%–10 % (v/v) DCM/MeOH gradient) to afford VU0500469 as a white solid (33 mg, 46%). 1H NMR (MeOH-d4, 400 MHz) *δ* 7.83 (d, J = 8.0 Hz, 2H), 7.64 (d, J = 8.0 Hz, 1H), 7.46 (d, J = 8.0 Hz, 2H), 7.24–7.18 (m, 2H), 4.37 (t, J = 7.2 Hz, 2H), 3.37 (t, J = 7.2 Hz, 2H), 3.00 (t, J = 8.0 Hz, 2H), 2.95 (t, J = 6.8 Hz, 2H), 2.78 (t, J = 8.0 Hz, 2H), 2.62 (s, 3H), 2.46 (s, 3H), 2.42 (s, 6H), 2.10 (t, J = 7.2 Hz, 2H); LCMS (ESI) tR: 1.01 min, m/z: 460.4 [M + H]+.

### N-(4-Methylbenzo[d]Thiazol-2-yl)-4-(Phenylsulfonyl)Butanamide (1)

To a solution of 4-(phenylsulfonyl) butanoic acid (68 mg, 0.3 mmol) in DMF (2 ml) was added DIPEA (0.21 ml, 1.2 mmol), followed by 1-[Bis(dimethylamino)methylene]-1H-1,2,3-triazolo[4,5-b]pyridinium 3-oxide hexafluorophosphate (HATU, 171 mg, 0.45 mmol). After stirring for 30 min at 0°C, 4-methylbenzo[d]thiazol-2-amine (49 mg, 0.3 mmol) was added to reaction mixture. The reaction mixture was stirred 12 h, diluted with water (3 ml), and the resulting precipitate collected and washed with water (3 ml × 10 ml). The crude amide 1) was obtained as a white solid (82 mg, 73%) and used for the next step without further purification. LCMS (ESI) tR: 1.06 min, m/z: 375.3 [M + H]+.

### 4-((4-Fluorophenyl)Sulfonyl)-N-(4-Methylbenzo[d]Thiazol-2-yl)Butanamide (2)

Amide 2 was obtained as a white solid (82 mg, 70%) following the procedure described above except using 4-((4-fluorophenyl)sulfonyl)butanoic acid (74 mg, 0.3 mmol) as the coupling partner. LCMS (ESI) tR: 1.07 min, m/z: 393.2 [M + H]+.

### N-(2-(Dimethylamino)Ethyl)-N-(4-Methylbenzo[d]Thiazol-2-yl)-4-(Phenylsulfonyl)Butanamide (VU0500458)

To a solution of N-(4-methylbenzo[d]thiazol-2-yl)-4-(phenylsulfonyl)butanamide (33 mg, 0.088 mmol) in THF (2 ml) at 0°C was added a solution of sodium bis(trimethylsilyl)amide (0.26 ml, 1 M solution in THF). After maintaining the reaction mixture at 0°C for 10 min, 2-bromo-N,N-dimethylethan-1-amine (41 mg, 0.18 mmol) in THF (0.5 ml) was added. The reaction mixture was allowed to warm to room temperature, maintained for 12 h, and quenched with saturated NH_4_Cl solution (5 ml). The quenched reaction was extracted with EtOAc (3 ml × 10 ml), and dried over MgSO_4_. The solvent was removed under reduced pressure and the residue was purified by column chromatography (0%–10 % (v/v) DCM/MeOH gradient) to afford VU0500458 as a yellow solid (16 mg, 41 %). 1H NMR (MeOH-d4, 400 MHz) *δ* 7.97 (d, J = 9.2 Hz, 2H), 7.75 (t, J = 8.4 Hz, 1H), 7.67 (d, J = 8.4 Hz, 2H), 7.65–7.63 (m, 1H), 7.25–7.18 (m, 2H), 4.42 (t, J = 7.2 Hz, 2H), 3.40 (t, J = 7.2 Hz, 2H), 2.98 (t, J = 6.8 Hz, 2H), 2.86 (t, J = 7.6 Hz, 2H), 2.63 (s, 3H), 2.48 (s, 6H), 2.14 (q, J = 7.6 Hz, 2H); LCMS (ESI) Rt 0.97 min, m/z: 446.4 [M + H]+.

### N-(2-(Dimethylamino)Ethyl)-4-((4-Fluorophenyl)Sulfonyl)-N-(4-Methylbenzo[d]Thiazol-2-yl)Butanamide (VU0916219)

VU0916219 was prepared using same method with VU0500458. 1H NMR (MeOH-D4, 400 MHz) *δ* 8.01 (dd, J = 8.8, 5.2 Hz, 2H), 7.65 (d, J = 7.2 Hz, 1H), 7.38 (t, J = 8.4 Hz, 2H), 7.27–7.19 (m, 2H), 4.4 (t, J = 7.2 Hz, 2H), 3.40 (t, J = 7.2 Hz, 2H), 3.00 (t, J = 6.0 Hz, 2H), 2.88 (t, J = 7.6 Hz, 2H), 2.63 (s, 3H), 2.50 (s, 6H), 2.15 (t, J = 7.6 Hz, 2H); LCMS (ESI) Rt 0.98 min, m/z: 464.3 [M + H]+.

## Results and Discussion

### Discovery of KCC2 Potentiators by High-Throughput Screening

To identify small-molecule KCC2 potentiators, we developed a high-throughput screening (HTS)-compatible KCC2 activity assay using a monoclonal HEK-293 cell line inducibly expressing KCC2 and constitutively expressing the Cl^−^ sensor SuperClomeleon (Grimley et al., 2013). In this assay, changes in SuperClomeleon fluorescence, reflective of changes in [Cl^−^]_i_, were used as a surrogate for measuring KCC2 activity (Figure 1A). Note that our Cl^−^ efflux assay measures the effect of the compounds immediately following their addition to the cell, enabling detection of fast-acting modulators. Throughout this manuscript, data are normalized to the Förster resonance energy transfer (FRET) ratio between the Cl^−^-sensitive YFP and Cl^−^ insensitive CFP (YFP/CFP ratio) of SuperClomeleon, where an increase in this ratio is interpreted as an increase in KCC2 activity. All of the activity assay data in HEK-293 cells reported in the manuscript were obtained at 37°C due to our observation that many of the compounds tested, in particular those from the class we focus on in the present studies, exhibit strong temperature dependence within this cell line.

To establish that the Cl^−^ flux assay was suitable for HTS, first we determined that the Cl^−^ flux assay reports KCC2 activity by demonstrating that the assay responds to known KCC2 modulators including indirect-acting KCC2 potentiators as well as a direct-acting KCC2 inhibitor (Supplementary Figure S1A), and that the observed responses to these modulators are dependent on KCC2 expression (Supplementary Figures S1B,C). Further, none of the compounds affected Cl^−^ flux in SuperClomeleon-expressing cells that had not be transfected with KCC2. After establishing that the Cl^−^ flux assay is capable of measuring KCC2 activity, we established that the assay was suitable for HTS by demonstrating highly reproducible separation between assay signal in vehicle-treated and KCC2 potentiator-treated wells. Our results show highly reproducible separation between potentiator- and control-treated cells on a plate-by-plate and day-by-day basis as quantified using the Z′ method (Zhang et al., 1999). For Z′ value calculation, we treated every other well on the 384-well plate with either vehicle or WNK463 (Yamada et al., 2016), a With-No-Lysine (WNK) kinase inhibitor that indirectly potentiates KCC2. These experiments yielded a mean Z’ value of 0.69 ± 0.03 (Figures 1B,C), consistent with a well-to-well and experiment-to-experiment separation of vehicle and KCC2 potentiator-treated wells suitable for HTS.

**FIGURE 2:**
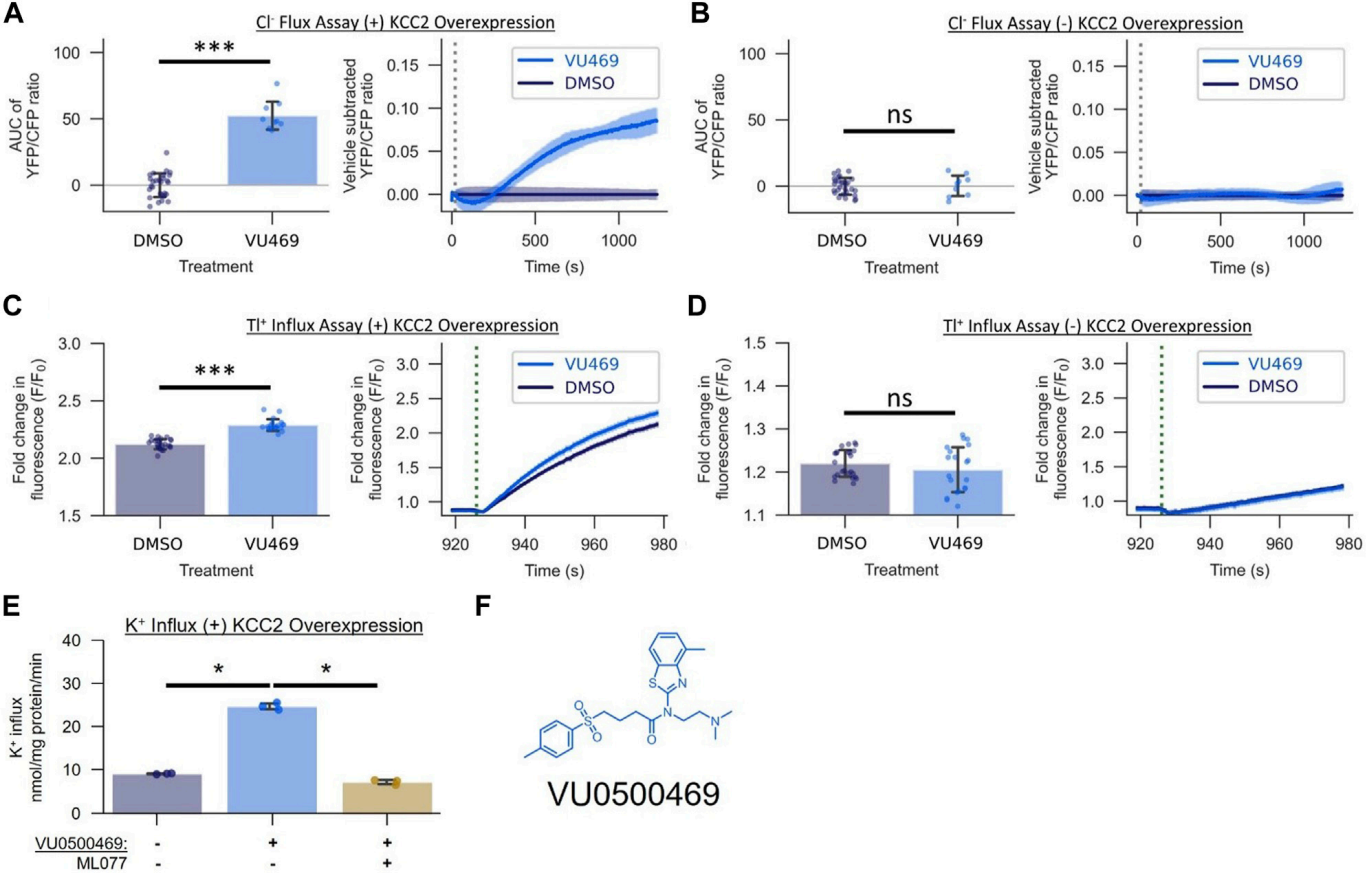
VU0500469 (VU469) potentiates KCC2-mediated Cl^−^ transport across mechanistically distinct assays of KCC2 activity in HEK-293 cells. **(A,B)** Effect of 15 µM VU0500469 on Cl^−^ flux in the presence or absence of KCC2 overexpression (*n* > 9). **(A)** Cl^−^ Flux Assay in cells overexpressing KCC2. Left: AUC of the YFP/CFP Cl^−^ efflux ratio. Right: Vehicle control subtracted and averaged traces of the YFP/CFP Cl^−^ efflux ratio. Dotted line represents time of compound addition. **(B)** Cl^−^ flux assay in cells lacking KCC2 overexpression. **(C,D)** Effect of 15 µM VU0500469 on Tl^+^ influx in the presence or absence of KCC2 overexpression (*n* > 20). **(C)** Tl^+^ influx in cells overexpressing KCC2. Left: maximum fold increase in Tl^+^-sensitive dye fluorescence. Right: fold increase in Tl^+^-sensitive dye fluorescence over time. Dotted line represents time point of Tl^+^ addition. **(D)** Tl^+^ influx in cells lacking KCC2 overexpression. **(E)** Effect of 30 µM VU0500469 on K^+^ influx in cells overexpressing KCC2 in the absence or presence of KCC2 inhibitor ML077 (*n* = 3). **(F)** VU0500469 structure. All experiments conducted at 37°C. Error bars represent SD. Statistical significance calculated by Welch’s t-test **(E)** and Mann–Whitney U test **(A–D)**: * = *p* < 0.05, *** = *p* < 0.001, ns = *p* > 0.05.

Having demonstrated that the Cl^−^ flux assay was suitable for HTS, we used it to screen ∼23,000 small molecules from the Vanderbilt Discovery Collection (Figure 1D, Supplementary Figure S1D). Compounds that promoted an apparent decrease in [Cl^−^]_i_ with a Z-score > 3 compared to vehicle-treated controls were selected for further testing. The compounds that displayed activity in cells expressing KCC2 but not cells lacking KCC2 expression were considered verified hits. Of the verified hits we discovered, VU0500469 (Figure 2F) stood out based on its unique pharmacological profile which is described in detail below (Figure 2).

### The Activity of VU0500469 Depends on the Expression of KCC2 and is Consistent Across Mechanistically Distinct Assays of KCC2-mediated Ion Transport

To establish that the activity of VU0500469 in the Cl^−^ efflux assay was dependent on the expression of KCC2, we tested it in HEK-293 cells in the presence and absence of KCC2 expression. VU0500469 enhanced Cl^−^ efflux compared to DMSO vehicle alone in cells expressing the cotransporter but not in cells lacking KCC2 expression (Figures 2A,B). To further evaluate VU0500469, we tested its activity in two mechanistically distinct KCC2 assays. We used the Tl^+^ influx assay which utilizes Tl^+^, a K^+^ congener, and a Tl^+^-sensitive fluorescent indicator (Weaver et al., 2004; Delpire et al., 2009) to report KCC2-mediated Tl^+^ influx. As seen in Figures 2C,D, cells expressing KCC2 demonstrate a significantly larger (*p* < 0.001) Tl^+^ uptake than cells lacking KCC2, and VU0500469 further enhanced this Tl^+^ influx in cells expressing the cotransporter. We also used radioactive ^83^Rb^+^ uptake to measure KCC2-mediated K^+^ influx (Xie et al., 2020) in KCC2 expressing HEK-293 cells. As seen in Figure 2E, 30 µM VU0500469 markedly stimulated K^+^ influx (*p* < 0.05), an effect that was abolished by the addition of a maximally effective concentration of ML077, a selective KCC2 inhibitor (Delpire et al., 2012).

### Improvement of the Pharmacological Properties of VU0500469 to Yield VU0916219

**FIGURE 3:**
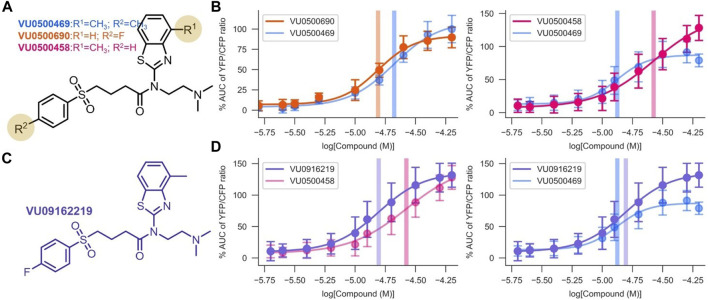
Development of VU0916219, a KCC2 potentiator with improved activity. Experiments conducted at 37°C, using the Cl^−^ Flux Assay in HEK-293 cells overexpressing KCC2. Data are normalized to maximum VU0500469 activity being 100%. **(A)** Structures of the most potent and efficacious VU0500469 analogs. **(B)** Comparison of VU0500469 dose-response curve to the most potent (left panel) and efficacious (right panel) compounds. **(C)** Structure of VU0916219. **(D)** Comparison of VU0916219 to the most efficacious compound (left panel), and VU0500469 (right panel). (n ≥ 6), error bars represent SD.

While we were encouraged by the first-in-class KCC2 potentiator activity of VU0500469, the compound exhibited lower potency (EC_50_, 14.2 ± 0.7 µM; Figure 3B) than desired. Starting with VU0500469, we sought to improve potency by further screening our in-house compound library and through direct synthetic modification of the VU0500469 scaffold (Figure 3, Supplementary Figure S2). We began by screening 200 compounds from the Vanderbilt Discovery Collection with structural similarity to VU500469 to develop a preliminary structure-activity relationship (Supplementary Figure S2A). Structural similarity to VU0500469 was quantified by Tanimoto coefficient (Tc) calculation after chemical fingerprinting (Peltason and Bajorath, 2008) of the Vanderbilt Discovery Collection. Compounds with the highest Tc were screened using the Cl^−^ flux assay in the presence and absence of KCC2 expression. From this screen, we discovered inactive analogs with high structural similarity to VU0500469, e.g., VU0500693 (Tc = 0.82) and VU0500849 (Tc = 0.79), which were utilized as inactive control compounds (Supplementary Figures S2B,C). The data demonstrate that changes to the structure of compounds closely related to VU0500469 can result in considerable changes in activity. Importantly, we discovered multiple KCC2-dependent Cl^−^ efflux potentiators with either improved potency, e.g. VU0500690 (Tc = 0.87), or improved efficacy, e.g. VU0500458 (Tc = 0.99) relative to VU0500469 (Figures 3A,B). We also discovered VU0500469 analogs that, while sharing the central benzothiazole moiety and retaining KCC2 potentiator activity, had appreciable differences in structure through the rest of the molecule. This is the case for VU0500789 (Tc = 0.73) and VU0496374 (Tc = 0.55) (Supplementary Figures S2D,E). From these data, we conclude that VU0500469 analogs can tolerate structural changes while maintaining an ability to potentiate KCC2 activity.

To confirm that the activities of VU0500469 and VU0500458 were in fact due to compounds with the structures as drawn, we resynthesized VU0500469 (Supplementary Scheme S1) and VU0500458 (Supplementary Scheme S2), and demonstrated that these resynthesized compounds (Figures 3B,D) retained the same activity in the Cl^−^ flux assay as the stocks from the Vanderbilt Discovery Collection (Figure 2A).

To improve upon the pharmacological properties of VU0500469, we sought to combine the fluorophenyl group of VU0500690 (the most potent compound discovered, Figures 3A,B) with the 4 methyl-benzthiazole from VU0500458 (the most efficacious compound. Figures 3A,B). We synthesized VU0916219 using a similar synthetic route to VU0500458 (Supplementary Scheme S2). As seen in Figure 3D, VU0916219 matched the efficacy of VU0500458 and increased in potency (EC_50_, 17.1 ± 1.2 µM vs. 28.8 ± 4.2 µM). While the potency of VU0916219 was still slightly lower than VU0500469 (EC_50_, 17.1 ± 1.2 µM vs. 14.2 ± 0.7 µM), the >40% increase in efficacy relative to VU0500469 resulted in an overall increase in KCC2 activity. These data establish a preliminary SAR for VU0500469-like compounds and indicate that the VU0500469 structural class is a suitable starting point for future medicinal chemistry efforts.

### VU0500469 Exhibits a Unique Pharmacological Profile Relative to Known KCC2 Potentiators

**FIGURE 4:**
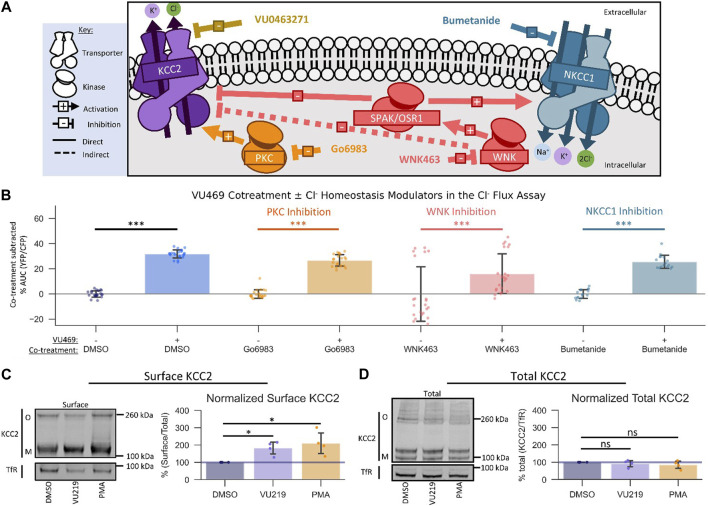
VU0500469 potentiates KCC2 by a unique mechanism. All experiments conducted using the Cl^−^ flux assay in HEK-293 cells overexpressing KCC2 at 37°C, unless otherwise noted. **(A)** Schematic depicting investigated small molecule targets known to regulate Cl^−^ homeostasis. **(B)** Co-treatment of modulators of Cl^−^ homeostasis from **(A)** with or without VU0500469 (*n* ≥ 16). **(C)** Left: representative Western blot of biotinylated surface fraction from cells treated with vehicle (DMSO), VU0916219 (VU219), or the positive control phorbol 12-myristate 13-acetate (PMA). Band intensity was normalized to Transferrin Receptor (TfR) levels. O, oligomeric KCC2, M, monomeric KCC2. Right: quantification of Surface KCC2/Total KCC2 normalized to TfR levels. **(D)** Left: representative Western blot of total protein fraction from cells treated as in **(C)**. Right: quantification of total KCC2 normalized to TfR levels. (*n* = 4) Error bars represent SD. Statistical significance calculated by Mann–Whitney U test **(B)** or paired t*-*test **(C,D)**: * = *p* < 0.05, *** = *p <* 0.001.

Confident that the activity of VU0500469 was KCC2-dependent, we next sought to evaluate whether VU0500469-like compounds act through an established KCC2 regulatory mechanism, or a novel one (Figure 4, Supplementary Figure S3).

There are many signaling pathways that indirectly influence the activity of KCC2 (Medina et al., 2014). Based on the rapid onset of VU0500469’s ability to potentiate Cl^−^ efflux, we reasoned that VU0500469 might act through one of three Cl^−^ homeostasis effectors known to be expressed in HEK-293 cells: PKC (Lee et al., 2007), WNK-SPAK/OSR1 (Friedel et al., 2015), and NKCC1 (Delpire et al., 2009) (Figure 4A). To test if VU0500469 affected these regulatory pathways, we first used a pharmacological co-treatment approach, together with the Cl^−^ flux assay, in KCC2-expressing HEK-293 cells. We observed that 20 µM VU0500469 retained its ability to potentiate Cl^−^ efflux (Figure 4B, Supplementary Figure S3A; *p* < 0.001) despite treating cells with saturating inhibitory concentrations of either PKC (Go6983) or WNK (WNK463) (Supplementary Figures S3B,C), suggesting that VU0500469 acts independently of these regulatory mechanisms. In addition, VU0500469 did not affect the flux of K^+^ in native HEK-293 cells with or without addition of bumetanide, indicating that the compound does not affect NKCC1 activity. Furthermore, we reasoned that VU0500469 acts by a distinct mechanism relative to compounds which increase KCC2 activity on the timescale of hours to days, such as CLP257 (Gagnon et al., 2013), owing to its rapid onset on the scale of minutes, and that VU0500469 works independent of pathways that increase KCC2 expression, such as KCC2 expression enhancing compounds (KEECs) (Tang et al., 2019), because VU0916219, our most active analog of VU0500469, does not alter total KCC2 protein expression (Figure 4D).

An established mechanism to increase the amount of KCC2 ion transport activity is by increasing the surface expression of KCC2 (Lee et al., 2007). To determine if VU0500469-like compounds were acting via this mechanism, we quantified KCC2 surface levels using surface biotinylation (Friedel et al., 2017) in response to compound treatment. We isolated surface fractions using streptavidin affinity purification from HEK-293 cells expressing KCC2, and we subsequently quantified surface and total KCC2 expression levels by immunoblot. Treatment with either 100 nM phorbol 12-myristate 13-acetate (PMA), a PKC agonist previously shown to increase KCC2 surface expression (Lee et al., 2007), or 60 μM VU0916219, our most active analog of VU0500469 (Figure 3C, Tc = 0.82), caused a statistically significant increase in KCC2 surface levels relative to vehicle-treated controls (Figure 4C; *p* < 0.05). In contrast, total KCC2 levels were statistically indistinguishable from vehicle-treated controls for both PMA and VU0916219 (Figure 4D; *p* > 0.05). Taken together, these data are consistent with the conclusion that the VU0500469-like compounds increase KCC2 surface expression without affecting total KCC2 expression.

### VU0500469 Prevents Synchronized Ca^2+^ Oscillations in Neuronal-Glial Co-Cultures

**FIGURE 5:**
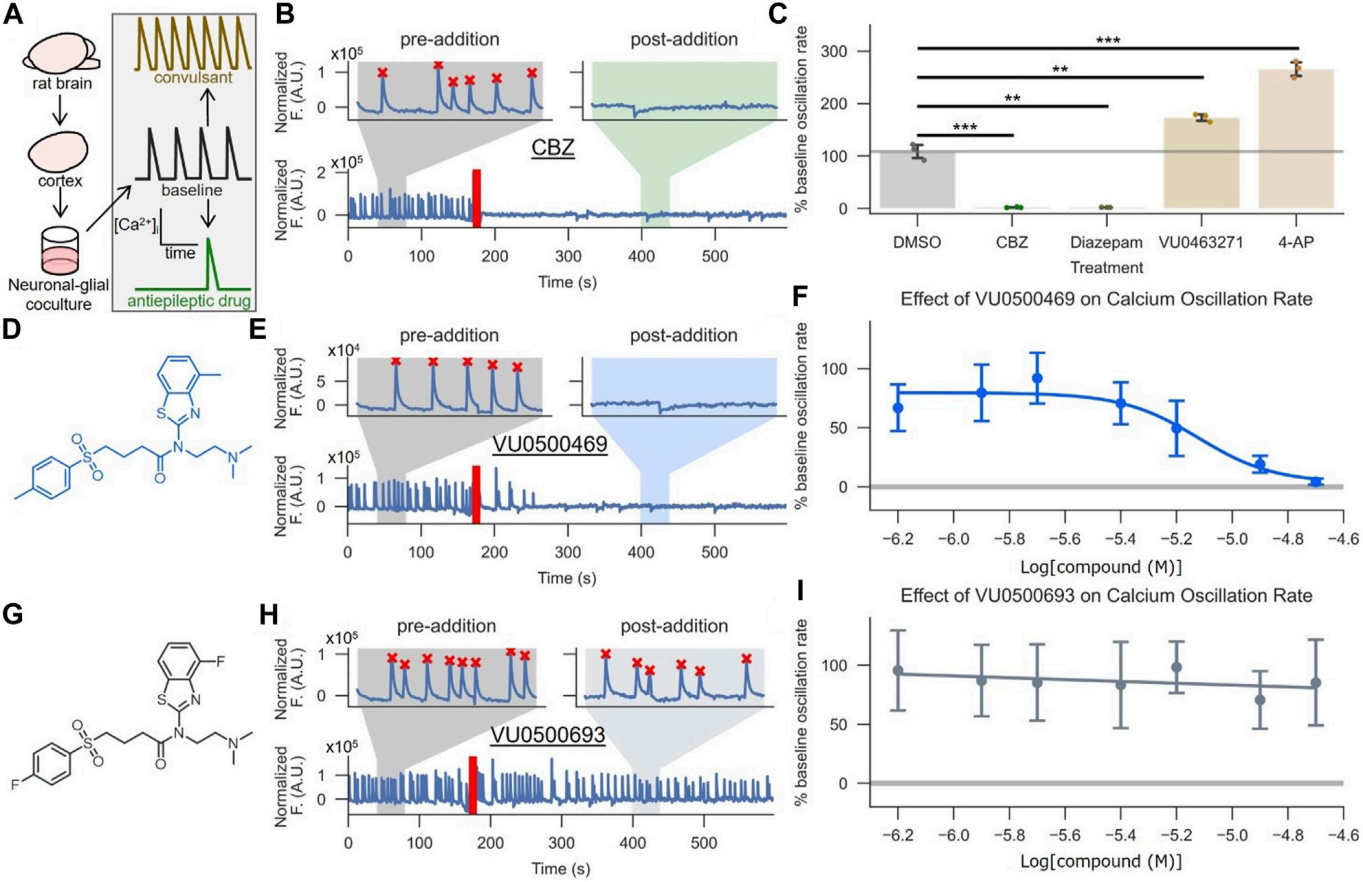
VU0500469 inhibits seizure-like, synchronous Ca^2+^ oscillations in cultured rat cortical neurons. **(A)** Cartoon description of the synchronous Ca^2+^ oscillation assay, which uses neuronal Ca^2+^ oscillations from high density neuronal-glial cocultures to model epileptiform activity. **(B,C)** Validation of the synchronous neuronal Ca^2+^ oscillation assay’s ability to report anti-epileptic and KCC2-dependent activity. **(B)** Example Ca^2+^ oscillation trace treated with the antiepileptic drug carbamazepine (CBZ). Segments before and after compound addition are highlighted to better show neuronal Ca^2+^ oscillations in the absence and presence of compound, respectively. The time point of compound addition denoted by the red line. Oscillations recognized by a spike counting algorithm are marked by a red “X.” **(C)** Quantified % pre-addition oscillation rate for CBZ, the GABA_A_ positive allosteric modulator diazepam, vehicle control, the convulsant 4-AP, and the KCC2 inhibitor VU0463271 (*n* = 3). **(D–F)** VU500469 dose-dependently prevents seizure-like Ca^2+^ oscillations. **(D)** VU0500469 structure. **(E)** Example trace treated with 20 μM VU0500469, annotated as in **(B)**. **(F)** Dose-dependent effect of VU0500469 on Ca^2+^ oscillation rate (*n* = 10). **(G–I)** VU0500469, a structurally related analog of VU0500469 without significant activity in the Cl^−^ flux assay, does not affect seizure-like Ca^2+^ oscillations. **(G)** VU0500469 structure. **(H)** Example trace treated with 20 μM VU0500469, presented as in **(B)**. **(I)** Dose-dependent effect of VU0500469 on % pre-addition oscillation rate. Error bars represent SD. (*n* = 10). Statistical significance calculated by one sample t-test (6B, DMSO v. diazepam) and Welch’s t-test for all other samples: ** = *p <* 0.01, *** = *p <* 0.001.

To assess the activity of VU0500469 on KCC2 in a more native system and investigate if this chemical class had activity on a seizure-like process, we utilized a neuronal-glial co-culture ‘seizure’ model (Pacico and Mingorance-Le Meur, 2014) (Figure 5A). This model is based on the observation that high-density neuronal-glial rat cortical cultures undergo synchronous Ca^2+^ oscillations that mirror synchronous electrical activity during seizure events. The rate of these Ca^2+^ oscillations is reduced when the cultures are treated with antiepileptic drugs, such as carbamazepine (CBZ) (Figures 5B,C; *p* < 0.01), and elevated when treated with convulsants, such as 4-aminopyridine (4-AP) (Figure 5C; *p* < 0.001).

To validate the suitability of this system for modeling KCC2 activity during seizure-like events, we demonstrated that 1) KCC2 was expressed in neuronal populations using immunofluorescence (Supplementary Figure S4A), and 2) KCC2 was functional in these neurons by demonstrating that selective pharmacological inhibition of KCC2 increased Ca^2+^ oscillation rates (Figure 5C; *p* < 0.01), while pharmacological potentiation of GABA_A_ receptors, which relies on the Cl^−^ gradient established by KCC2, decreased Ca^2+^ oscillation rates (Figure 5C; *p* < 0.01) (Schulte et al., 2018).

Treatment of these neuronal-glial co-cultures with VU0500469 caused a dose-dependent decrease in Ca^2+^ oscillations (Figures 5D–F) while a closely related analog, VU0500693, that showed negligible activity in the Cl^−^ flux assay (Supplementary Figure S2B) failed to decrease Ca^2+^ oscillation rate (Figures 5G–I). Moreover, there was a statistically significant correlation between increased KCC2 activity in the HEK-293 cell-based Cl^−^ flux assay and a decrease in neuronal Ca^2+^ oscillation rate (Supplementary Figure S4B, *p* < 0.05). Collectively, these results support the conclusion that VU0500469 potentiates KCC2 activity in neurons and in doing so, decreases seizure-like activity.

## Conclusion

We have discovered and performed preliminary characterization of a new class of small-molecule KCC2 potentiator, and we have determined that this class can decrease seizure-like events in an *in vitro* model of epilepsy. The discovery of a new KCC2 potentiator class is significant because these compounds provide a new set of tools to further the investigation of KCC2’s therapeutic potential in a host of neurological disorders with tremendous unmet medical need. Our discovery that the VU0500469 class prevents *in vitro* seizure-like events further validates the antiepileptic promise of KCC2 potentiation, and provides preliminary evidence that pharmacological KCC2 potentiation, alone, is sufficient to produce an antiepileptic effect. These findings could help lay the foundation for the development of new treatments for epilepsy. While promising, our current best compounds do have limitations, most notably in terms of their potency for potentiating KCC2. Future work will focus on improving the potency of compounds in the VU0500469 class, more fully evaluating the selectivity of VU0500469-like compounds across a broad range of targets and establishing a more complete understanding of the mechanism by which VU0500469-like compounds potentiate KCC2 activity.

## Data Availability

The raw data supporting the conclusion of this article will be made available by the authors, without undue reservation.
