# Supplementary material for: Discovery of Small Molecule KCC2 Potentiators Which Attenuate In Vitro Seizure-Like Activity in Cultured Neurons
Source: Front Cell Dev Biol. 2022 Jun 24;10:912812. doi: 10.3389/fcell.2022.912812 (PMC9263442; doi:10.3389/fcell.2022.912812)

Discovery of Small Molecule KCC2 Potentiators Which Attenuate *In Vitro* Seizure-Like Activity in Cultured Neurons

Supplementary Figures

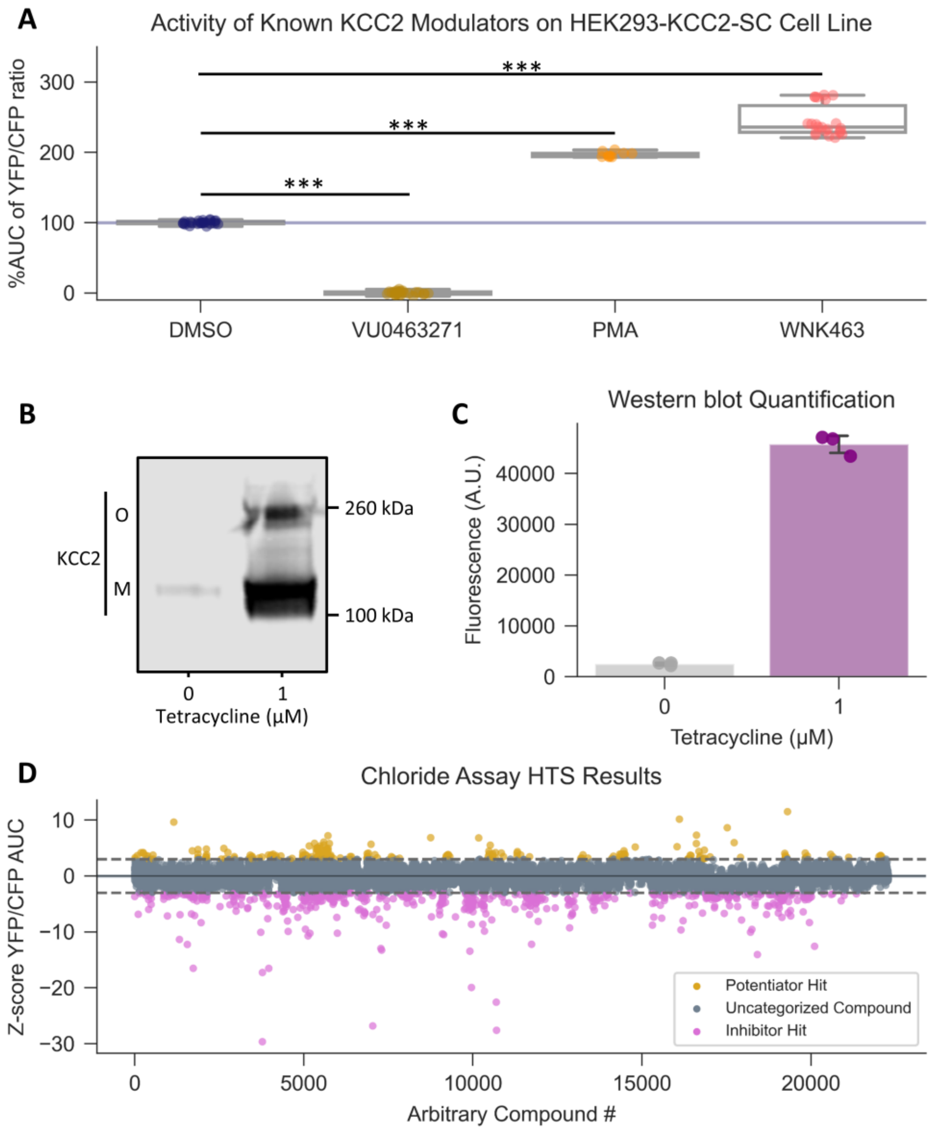

Figure S1. The cell line used for screening exhibits KCC2-dependent pharmacology, and induction-dependent expression of KCC2. (**A**) Activity of a panel of known KCC2 modulators on YFP/CFP ratio in the KCC2- and SuperClomeleon-expressing HEK-293 cell line used for high-throughput screening, 4p2.F7 [$n \geq 9$]. (**B**) Detection of KCC2 in HEK-293-KCC2-SC cell line [4p2.F7] lysates by immunoblotting, with or without induction by tetracycline. (**C**) Quantification of signal from (B) [$n = 3$]. (**D**) Unsorted HTS results with potentiator and inhibitor hits labeled. Solid line represents vehicle-treated mean. Dotted lines represent 3*SD above or below the vehicle-treated mean. All experiments ran at 37°C. *** = $p < 0.001$ by Mann Whitney U test. Error bars represent SD.

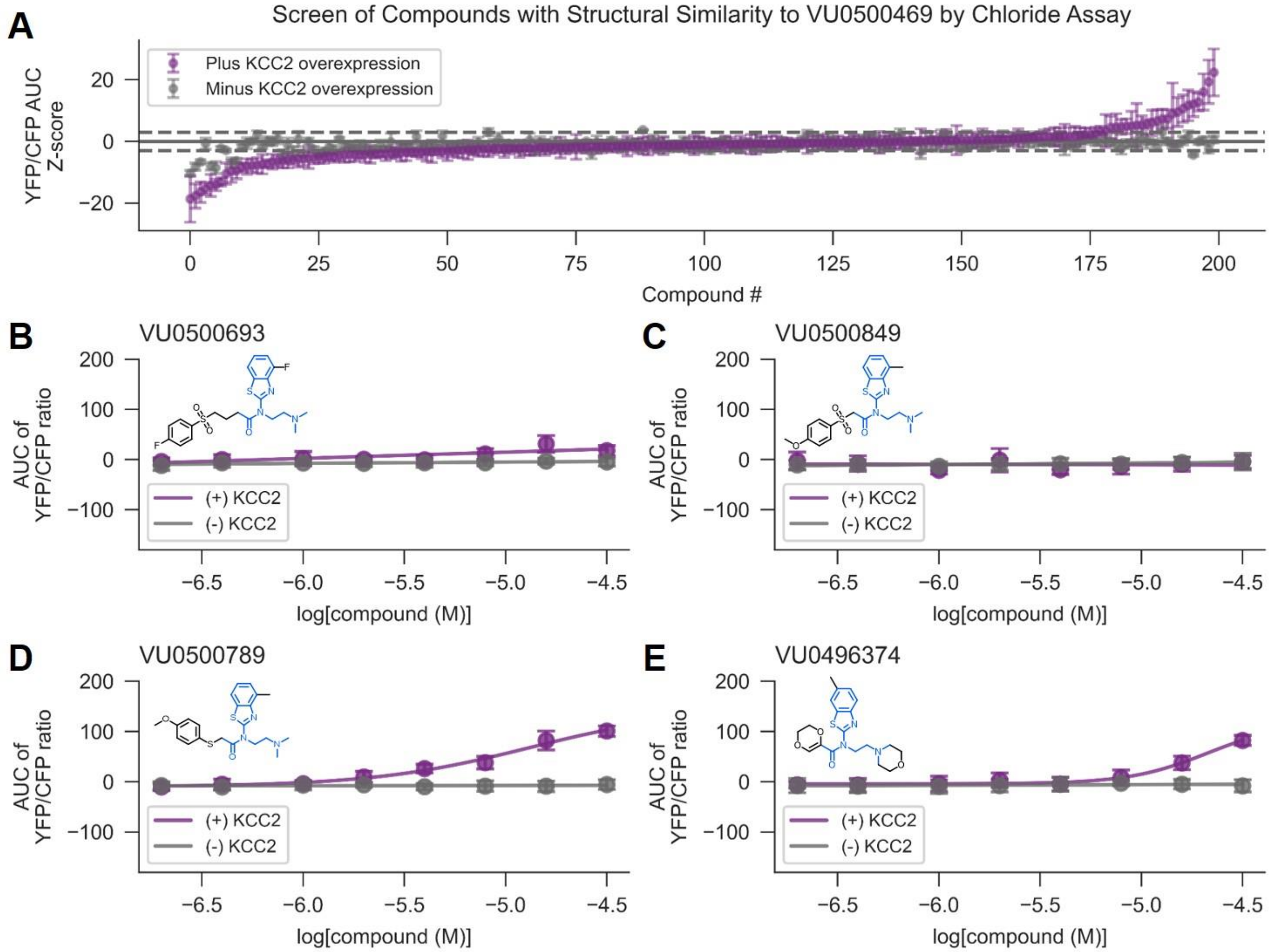

Figure S2. Representative VU0500469 analogs with multiple activity modes on KCC2. Data generated in HEK-293 cells overexpressing KCC2 at 37°C using the Cl^- flux assay\. (**A**) Screening results of 200 compounds with similar structure to VU0500469 ± KCC2 overexpression. (**B**-**E**) Dose response curves ± KCC2 overexpression for VU0500469 analogs with distinct activity and structure. *Inset*: structures of compounds used in the dose response curves, with similar structural regions between compounds highlighted. [n $\geq$ 4]. Error bars represent SD.

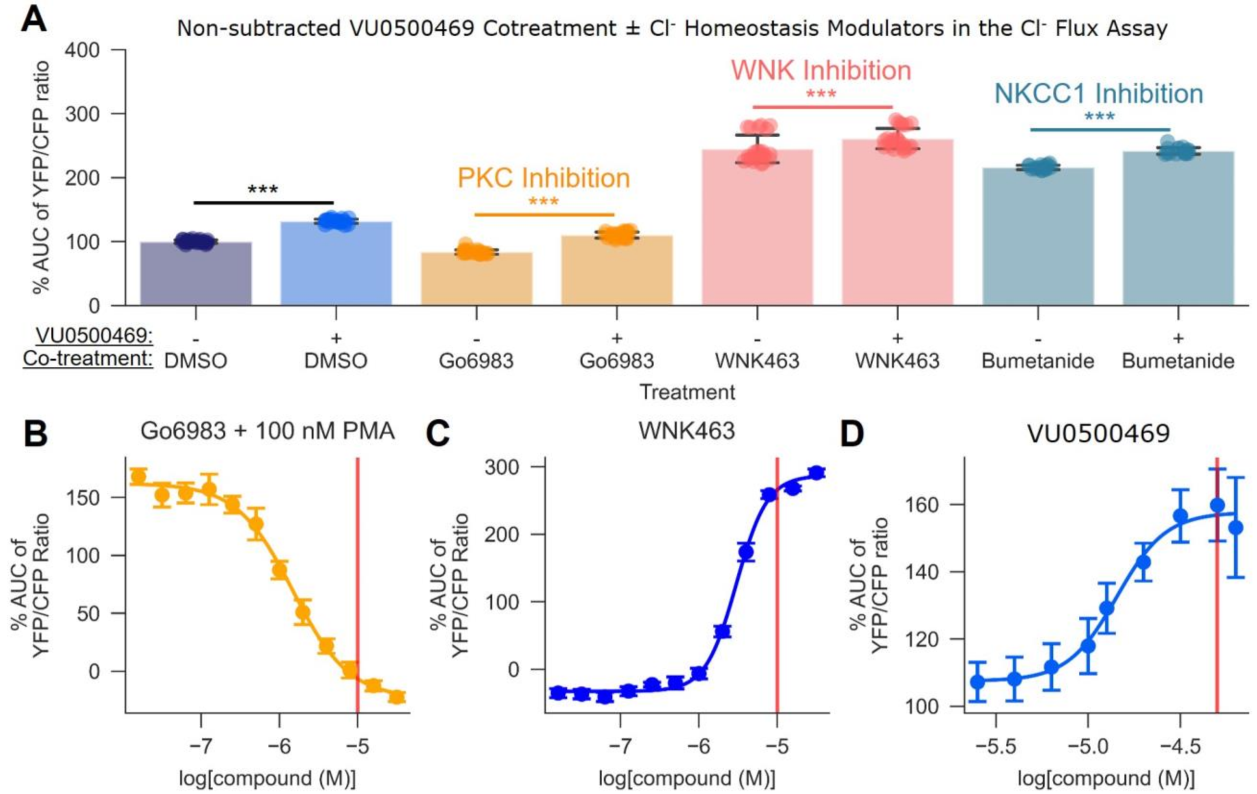

Figure S3. Additional validation data for unique KCC2 potentiation mechanism by VU0500469. All data generated in HEK-293 cells overexpressing KCC2 at 37°C, unless otherwise noted. (***A***) Data from VU0500469 co-treatment experiments without control subtraction. Experiment used 10 µM of indicated modulator of Cl^- homeostasis ± 20 µM VU0500469. *** $p < 0.001$, Mann Whitney *U* test. (***B-D***) Dose-response curves for PKC inhibitor Go6983, WNK inhibitor WNK463, and VU0500469 to calculate the dose needed for maximal efficacy in the Cl^- flux assay. Red line indicates maximum concentration used in co-treatment experiments. [$n \geq 8$].

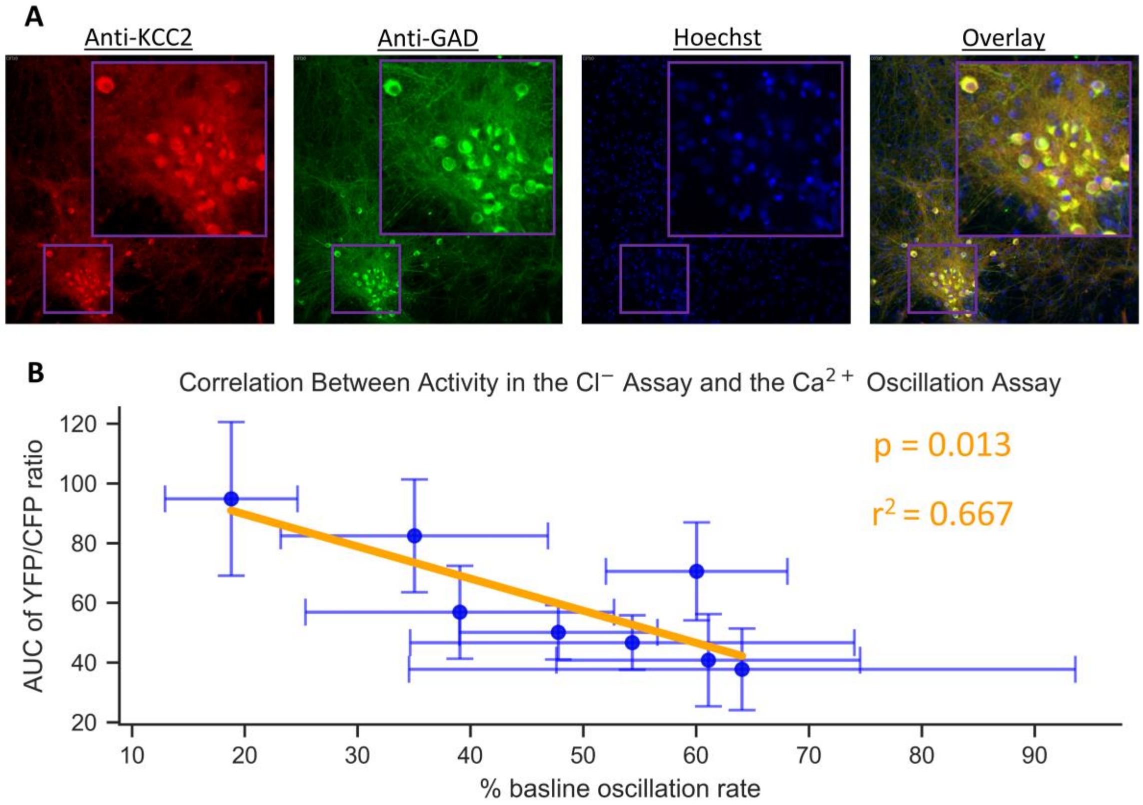

Figure S4. Additional validation of compound activity on KCC2 in the synchronous neuronal Ca^{2+} oscillation assay. (**A**) KCC2 immunoreactivity colocalizes with immunoreactivity from an inhibitory neuronal marker, GAD. Representative immunofluorescence images from neuronal-glial cocultures used in the synchronous neuronal Ca^{2+} oscillation assay. (**B**) Plot of compound activity at 30 µM in the Cl^- flux assay against compound effect on % baseline oscillation rate at 6 µM in the neuronal Ca^{2+} oscillation assay [n = 4]. Error bars represent SD. p = 0.013, Wald Test with t-distribution of the test statistic; $r^2 = 0.667$.

Scheme S1: Synthesis of VU0500469

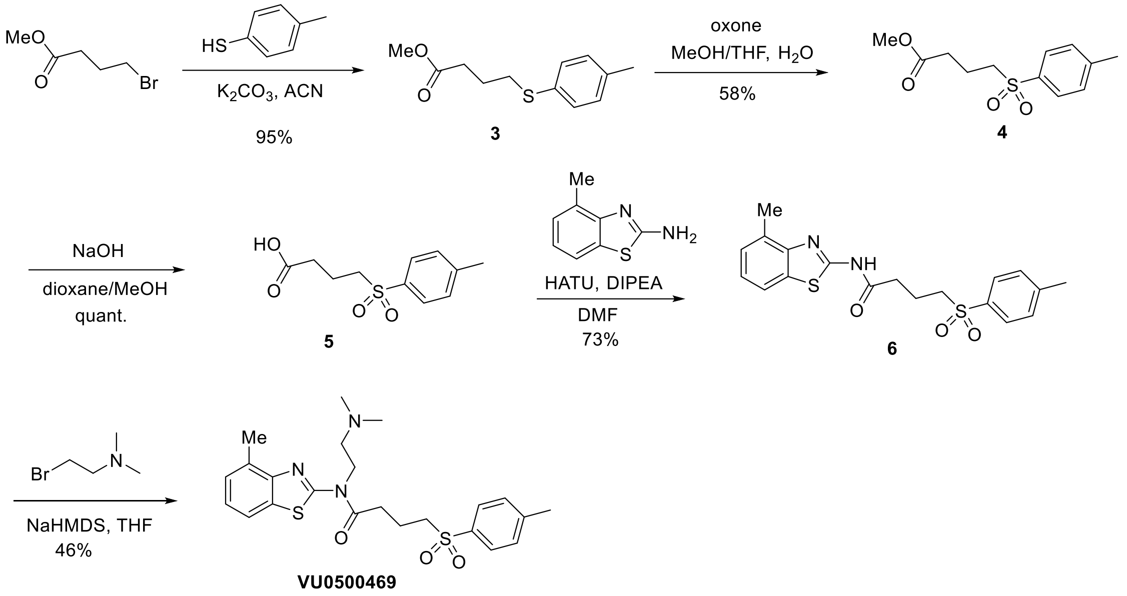

Scheme S2: Synthesis of VU0500458 and VU0916219.

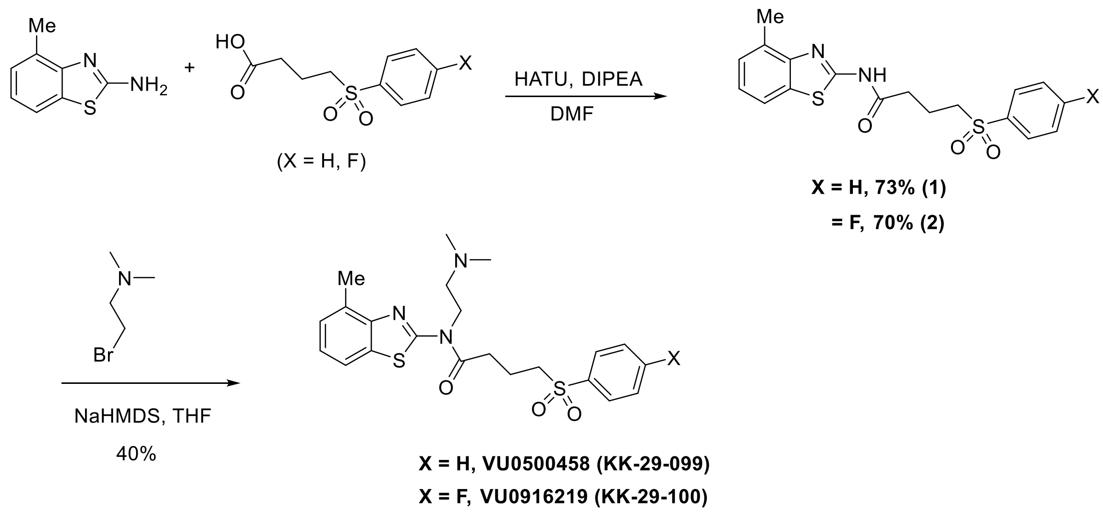

Supplement: Supplementary file 1 [file Image1.pdf]
